# The COVID-19 Pandemic Affects Male Patients With Chronic Spontaneous Urticaria More Than Female Patients

**DOI:** 10.3389/fimmu.2021.722406

**Published:** 2021-10-11

**Authors:** Huzeyfe Kulu, Mustafa Atasoy, Kemal Özyurt, Marcus Maurer, Atıl Avcı, Muhammet Reşat Akkuş, Ragıp Ertaş

**Affiliations:** ^1^ Urticaria Center of Reference and Excellence (UCARE), Chronic Skin Diseases Unit, Department of Dermatology, Kayseri City Hospital, Kayseri, Turkey; ^2^ Urticaria Center of Reference and Excellence (UCARE), Dermatological Allergology, Department of Dermatology and Allergy, Charité - Universitätsmedizin Berlin, Berlin, Germany

**Keywords:** chronic spontaneous urticaria, chronic skin diseases, COVID-19, psoriasis, pandemic (COVID19)

## Abstract

**Introduction:**

The COVID-19 pandemic dramatically disrupts health care for patients with chronic diseases including chronic spontaneous urticaria (CSU). As of now, it is unknown if the effects of the pandemic in CSU are different than in other chronic diseases. We also do not know, if different groups of CSU patients, for example female and male patients, are affected differently.

**Aim:**

To understand how CSU patients and subgroups are affected by the COVID-19 pandemic in their disease activity and control and treatment, using psoriasis as control.

**Patients and Methods:**

We analyzed 399 patients (450 visits) with CSU or psoriasis assessed during August 2019, i.e. before the pandemic, or August 2020, i.e. during the pandemic, for changes in disease activity, disease control, and the treatment they used, and how these changes are linked to age, gender, and disease duration.

**Results:**

Male but not female patients with CSU had markedly increased disease activity during the pandemic. CSU patients’ age or disease duration were not linked to changes. Male and female patients with psoriasis showed similar increases in disease activity and decreases in disease control. The rate of omalizumab treatment, during the pandemic, was unchanged in male patients and increased in female patients with CSU. The efficacy of omalizumab treatment, during the pandemic, was reduced in male patients but not female patients with CSU.

**Conclusion:**

Male but not female CSU patients, during the COVID-19 pandemic, show loss of disease control linked to loss of omalizumab efficacy. The reasons for this need to be investigated.

## Introduction

The COVID-19 pandemic caused by SARS-CoV-2 has caused ongoing challenges for health care systems across the globe^1^. One of them is the disruption of routine clinical care for patients with chronic diseases (1). Chronic diseases require continued monitoring, and patients are often in need of treatment adaptation. The COVID-19 pandemic, with lockdowns, travel restrictions, and a redistribution of health care resources towards testing and treating patients for COVID-19 has severely reduced the ability of patients with chronic diseases to obtain treatment and of physicians to provide it. This affects patients with various chronic diseases such as diabetes, chronic obstructive pulmonary disease, hypertension, asthma, cancer, depression, psoriasis and chronic urticaria (1–3).

Chronic urticaria (CU) is a common and disabling disease that manifests with pruritic wheals, angioedema, or both. The most common type of CU, chronic spontaneous urticaria (CSU), comes with highly fluctuating disease activity and is unpredictable in terms of when and where its signs and symptoms occur. Many environmental triggers such as stress and infections (and non-steroidal anti-inflammatory drugs that are used to treat them) can lead to increased disease activity, and they often do. Also, CSU is a disease that comes with impairment at work and in school, with sleep disturbance and sexual dysfunction (4), and can be hard to treat, as many patients do not achieve disease control with first and second line treatment, i.e. an antihistamine used at standard or higher than standard dose (5, 6). Psoriasis, like CSU, is a common, chronic, and disabling inflammatory skin disease. Unlike CSU, psoriasis manifests with stationary or progressing lesions, most commonly erythematous squamous plaques (7). Both CSU and psoriasis, in most patients, seriously reduce quality of life (6, 8).

Very recently, the COVID-CU study performed by the global network of Urticaria Centers of Reference and Excellence (UCAREs (9)) showed that the COVID-19 pandemic severely impairs patient care at urticaria specialist centers, markedly changes physician‐patient interactions, and affects how patients are treated. Its results also indicate that CU is not linked to severe COVID‐19, but often worsened by it (3, 10). The UCARE COVID-CU study investigated a limited number of CSU patients, few per country, across several countries affected differently by the pandemic, and it did not include controls (3). Because of the low number of patients included in the COVID-CU study, meaningful analyses of subpopulations of patients, e.g. male *vs* female or young *vs* old CU patients, were not possible. These limitations and the questions that emerged from the results of the COVID-CU study prompted the present study, which focused on the following questions: How is the care for patients with CSU and the management of their disease affected by the pandemic as compared to that of patients with other chronic inflammatory skin diseases, e.g. psoriasis? What are the effects of the pandemic on the levels of disease activity and on the treatment in patients with CSU as compared to patients with psoriasis? How has the pandemic affected the use of biologics in patients with CSU and psoriasis? Does the pandemic affect different CSU patients differently?

To address these questions, we chose a unique approach and made use of the fact that our UCARE is linked to the chronic skin diseases unit (CSDU) of our dermatology department, which provides outpatient services for patients with chronic inflammatory dermatoses including psoriasis (11). We selected the month of August of the past year, 2020, as our observation period, i.e. a time with intermediate numbers of SARS-CoV-2 infections and new COVID-19 cases in our country, after the first wave earlier that year. Our control observation period was the same month, August, of the previous year, 2019, i.e. before the pandemic. With the data from these two observation periods, from a sizeable number of CSU and psoriasis patients treated at our UCARE and CSDU, we analyzed the impact of the pandemic on both patient cohorts and subpopulations in terms of their outpatient care, disease activity and treatments.

## Patients and Methods

### Study Design and Conduct

A total of 450 visits of 399 patients with CSU or psoriasis treated at our UCARE and CSDU during August of 2019 or August of 2020 were analyzed in this retrospective study. Of these 399 patients, 51 patients, 27 with CSU and 24 with psoriasis, were treated in both, August of 2019 and August of 2020. None of the patients analyzed had CSU and psoriasis.

Of the 450 patient visits, 184 (41%) were for CSU and 266 (59%) for psoriasis. In August of 2019, 113 patients with CSU visited our outpatient clinic, 83 of them female (73.5%). In August of 2020, 71 CSU patients, 50 of whom were female (70.4%), visited the outpatient clinic. The August 2019 and 2020 average age of CSU patients was 41.2 ± 14.2 years and 39.6 ± 11.7 years, respectively.

In August of 2019, a total of 215 patients with psoriasis visited the outpatient clinic, 121 of them female (56.3%). In August of 2020, 51 psoriasis patients, 24 of whom were female (47.1%), visited the outpatient clinic. The August 2019 and 2020 average age of psoriasis patients was 41.4 ± 16.0 years and 43.6 ± 16.8 years, respectively.

Informed consent was obtained from each patient who participated in the study. Approval for this study was obtained from the Ethics committee of Kayseri City Education and Research Hospital. We compared 2019 and 2020 numbers of outpatients seen during the month of August, in total and by disease, as well as patient demographic data. Patient consultations outside of these dates were not investigated in this study. No other criteria were used in the selection of patients.

### Patient Assessment

The demographic information of patients was obtained from the hospital data management system. The parameters examined in patients with CSU included age, gender, disease activity as assessed by use of the urticaria activity score (UAS), disease control as measured by the urticaria control test (UCT) and the treatment they used [antihistamine, omalizumab, other (cyclosporine, methotrexate, etc.)]. For 2019, UAS and UCT scores for 27 patients were missing, and also the treatment data for 23 of 27 CSU patients were not available in that period. In 2020, UAS and UCT scores were missing of only 1 patient.

The UAS consists of two questions, one on the intensity of itch and one on the number of wheals in the last 24 hours. It is scored from 0 to 6, with an itch score between 0 and 3 (0=none; 3=very severe) and a wheal score between 0 and 3 (0=none; 3=more than 50 wheals in last 24 hours) (5). The UCT measures disease control during the last 4 weeks; it consists of four questions, with a score between 0 and 4 assigned to every answer option. The scores for all 4 questions are summed up for the total UCT, which ranges from 0 (no control) to 16 (complete control) (12). A UCT score of 12 points or higher indicates well-controlled disease (5). In CSU patients, until August 2020, we had no patients who were positive for COVID-19 PCR. Since two of our patients had symptoms, they applied with the suspicion of COVID, but PCR was not positive.

The parameters examined in the psoriasis patients included in the study were their age, gender, disease activity as assessed with the psoriasis area and severity index (PASI), and the treatment they used (topical therapy, phototherapy, conventional treatments [acitretin, methotrexate, cyclosporine] and biological treatments [adalimumab, infliximab, certolizumab, ustekinumab, secukinumab, ixekizumab]). For 2019, treatment data were unavailable for 11 of 215 patients. In 2020, all psoriasis datasets were complete. The PASI is one of the most used tools for the assessment of psoriasis severity and combines the measurement of skin lesion severity and affected skin area in a total score that ranges from 0 (no disease) to 72 (maximal disease) (13). We categorized psoriasis patients with a total PASI score of 0 or 1, i.e. those with no or minimal disease, as having controlled disease and those with PASI >1 as having uncontrolled disease.

We did not have any vaccinated patients since there was no approved vaccine in the period up to the date we base our study on.

### Statistical Analyses

The collected data were analyzed, and comparisons for statistically significant differences between groups were made using the IBM SPSS 25 package program. For continuous variables, the data were shown as mean ± standard deviation or median (interquartile range, IQR) and for categorical variables as frequencies (percentiles). Descriptive analyzes, chi-square test and Student t-test or Mann-Whitney U test were used. P values of less than 0.05 were considered to indicate significant differences.

## Results

### During the Pandemic, Disease Activity in Male, but Not Female Patients With CSU Increases

**Figure 1:**
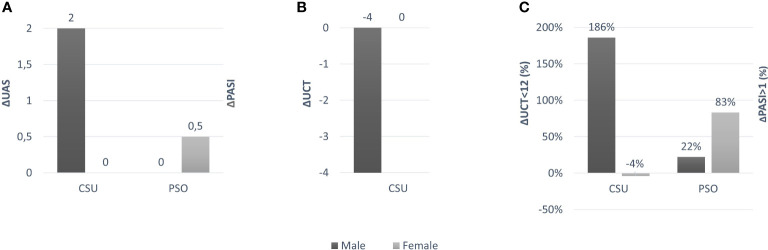
**(A)** Difference in UAS and PASI values for female and male patients between 2019 and 2020; **(B)** Difference in UCT value for female and male patients between 2019 and 2020; **(C)** Difference in% between UCT <12 and PASI> 1 values in female and male patients between 2019 and 2020.

Male patients with CSU had markedly increased disease activity during the pandemic (UAS: 3, 1-5) as compared to before the pandemic (UAS: 1, 0-2, p = 0.028, **Figure 1A** and **Table 1**). Male CSU patients, during the pandemic, also had lower levels of disease control (UCT: 11, 7-15) than before the pandemic (UCT: 15, 12-16; p = 0.029, **Figure 1B** and **Table 1**). Before the pandemic, 80% of male patients with CSU had well controlled disease, i.e. a UCT of 12 or more (**Table 1**). In 2020, the rate had dropped to 42.8% (p = 0.009; **Figure 1C** and **Table 1**). In contrast, disease activity in female patients with CSU was unchanged during the pandemic (UAS: 2, 1-4 in 2020 *vs* 2, 0-5 in 2019), as were levels of disease control (UCT: 12, 9-15 in 2020 *vs* 12, 6-16 in 2019) and rates of patients with well controlled disease (50.8% in 2019 *vs* 53.0% in 2020; **Figure 1** and **Table 1**). Unlike gender, CSU patients’ age and disease duration were not linked to increased disease activity during the pandemic.

**Table 1:** Number of outpatient visits, treatment and disease activity in female and male patients with psoriasis and CSU before and during the COVID-19 pandemic.

		Before the pandemic (2019)	During the pandemic (2020)	P value
**CSU outpatient visits**				**<0.0001** ^¶^
Female patients		83	50 (-40%)	0.736^¶^
Male patients		30	21 (-30%)	
All patients		113	71 (-37%)	
**Psoriasis outpatient visits**				
Female patients		121	24 (-81%)	0.274^¶^
Male patients		94	27 (-62%)	
All patients		215	51 (-77%)	
**Female patients with CSU**	On omalizumab, n (%)	21 / 63 (33.3%)	33 / 50 (66.0%)	**0.001^¶^**
	UAS, median (IQR)	2 (0-5)	2 (1-4)	0.622^§^
	UCT, median (IQR)	12 (6-16)	12 (9-15)	0.858^§^
	UCT≥12, n (%)	31 / 61 (50.8%)	26 / 49 (53.0%)	0.815^¶^
**Male patients with CSU**	On omalizumab, n (%)	13 / 27 (48.1%)	12 / 21 (57.1%)	0.159^¶^
	UAS, median (IQR)	1 (0-2)	3 (1-5)	**0.028^§^**
	UCT, median (IQR)	15 (12-16)	11 (7-15)	**0.029^§^**
	UCT≥12, n (%)	20 / 25 (80.0%)	9 / 21 (42.8%)	**0.009^¶^**
**All patients with CSU**	On omalizumab, n (%)	34 / 90 (37.8%)	45 / 71 (63.4%)	**< 0.0001** ^¶^
	UAS, median (IQR)	2 (0-4)	2 (1-4)	0.105^§^
	UCT, median (IQR)	12 (8-16)	11,5 (9-15)	0.302^§^
	UCT≥12, n (%)	51 / 86 (59.3%)	35 / 70 (50.0%)	0.245^¶^
**Female patients with psoriasis**	Biological therapy, n (%)	32 / 113 (28.3%)	11 / 24 (45.8%)	0.141^¶^
	PASI, median (IQR)	1 (0-2.0)	1.5 (0-4.35)	**0.030^§^**
	PASI≤1, n (%)	88 / 121 (72.7%)	12 / 24 (50.0%)	**0.028^¶^**
**Male patients with psoriasis**	Biological therapy, n (%)	25 / 91 (27.4%)	7 / 27 (25.9%)	0.276^¶^
	PASI, median (IQR)	1 (0-3.0)	1 (1.0-5.0)	**0.050^§^**
	PASI≤1, n (%)	57 / 94 (60.6%)	14 / 27 (51.9%)	0.414^¶^
**All patients with psoriasis**	Biological therapy, n (%)	57 / 204 (27.9%)	18 / 51 (35.3%)	0.060^¶^
	PASI, median (IQR)	1 (0-2.0)	1 (0.5-4.8)	**0.002^§^**
	PASI≤1, n (%)	145 / 215 (67.4%)	26 / 51 (51.0%)	**0.027^¶^**

CSU, Chronic Spontaneous Urticaria; IQR, interquartile range; PASI, Psoriasis Area and Severity Index; UAS, Urticaria Activity Score; UCT, Urticaria Control Test; ¶, Chi-squared test; §, Mann-Whitney U test.

Values in bold highlight statistically significant values.

As for patients with psoriasis, male and female patients, during the pandemic, showed similar and increased levels of disease activity, i.e. higher PASI scores, than before the pandemic (**Table 1**). During the pandemic, the rates of patients with minimal disease, i.e. PASI 1 or 0, dropped from 60.6% in 2019 to 51.9% in males and from 72.7% in 2019 to 50.0% in females (**Figure 1** and **Table 1**).

### The COVID-19 Pandemic Is Linked to Markedly Reduced Outpatient Visits of Both Male and Female Patients With CSU and Psoriasis

The number of monthly consultations of patients with CSU or psoriasis dropped from 328 before the pandemic to 122 (-63%) during the pandemic (**Table 1**). CSU outpatient visits went down to 71 per month during the pandemic as compared to 113 per month before the pandemic (-37%; -40% and -30% in female and male patients, respectively; **Table 1**). Outpatient visits of patients with psoriasis were reduced by 77% (-81% in female and -62% in male patients), from 215 per month before the pandemic to 51 per month during the pandemic, a significantly greater reduction than in CSU patients (p < 0.0001; **Table 1**).

### The Rate of Omalizumab Treatment, During the Pandemic, Is Unchanged in Male Patients and Increased in Female Patients With CSU

**Figure 2:**
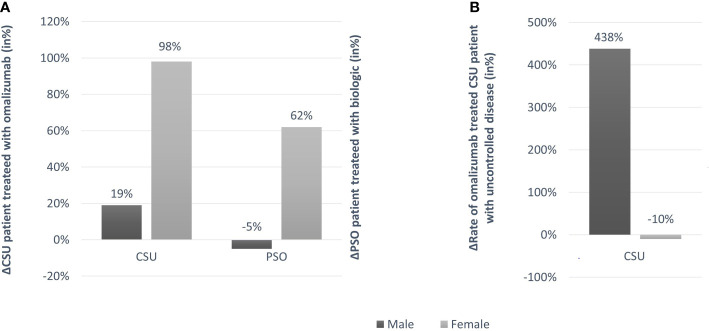
**(A)** Change rates (in%) of CSU patients treated with omalizumab and psoriasis patients treated with biologics, in 2019 and 2020; **(B)** Change rate (in%) of CSU patients treated with omalizumab with uncontrolled disease in 2019 and 2020.

With the decision taken by the Turkish Ministry of Health at the beginning of the pandemic period, all chronic patients would be able to take their medicines without applying to the hospital. Despite this decision, we identified patients who did not use their medication regularly. In male CSU patients using omalizumab, only 2 patients out of 13 could not use their medication regularly (15%) during the pandemic period. Similarly, in female CSU patients using omalizumab, 6 out of 33 patients could not take their medication regularly during the pandemic period (18%). The loss of disease control and increase in disease activity in male CSU patients during the pandemic was not due to a change in their rate of omalizumab treatment. Before the pandemic, 48.1% of male patients were treating with omalizumab, similar to during the pandemic, when the rate was 57.1% (**Table 1** and **Figure 2A**). Female patients with CSU showed increased rates of omalizumab treatment during the pandemic, 66.0% in 2020, up from 33.3% in 2019 (p=0.001; **Table 1** and **Figure 2A**). Overall, the use of omalizumab increased from 37.8%, in 2019, to 63.4%, in 2020.

In psoriasis, female patients also used biologics more frequently during the pandemic as compared to before the pandemic, and male patients also did not. Overall, the rate of psoriasis patients on biologic treatment increased to 35.3% from 27.9% during 2019 to 2020. Before the pandemic, 27.4% of male psoriasis patients were on biologic treatment, similar to during the pandemic, when the rate was 25.9%. Female patients with psoriasis showed increased and markedly higher rates of biologic treatment during the pandemic, 45.8% in 2020, up from 28.3% in 2019 (**Table 1** and **Figure 2A**).

### The Efficacy of Omalizumab Treatment, During the Pandemic, Is Reduced in Male Patients but Not Female Patients With CSU

Before the pandemic, 91.7% of male CSU patients treating with omalizumab had controlled disease, i.e. a UCT≥12. During the pandemic, CSU in male patients on omalizumab treatment was controlled in only 50% of patients (p=0.025). In contrast, female patients who treated with omalizumab had similar rates of controlled disease in 2019 and 2020, 63.2% and 66.7%, respectively (**Figure 2B**).

## Discussion

Our study shows, unexpectedly, that the current COVID-19 pandemic affects male and female patients differently, an effect not seen in psoriasis. Male patients experienced a drop in their rate of having CSU under control by more than 50% during the pandemic. This finding is explained, in part, by stagnant *vs* higher rates of omalizumab treatment and by markedly lower rates of response to omalizumab treatment in male *vs* female CSU patients during the pandemic. Why omalizumab is seemingly less effective in males during the pandemic is unknown. Several hypotheses are discussed below and should be tested in future studies.

That the COVID-19 pandemic comes with markedly reduced outpatient visits in patients with CSU confirms the results of the recent COVID-CU study (3). In our center, CSU patient visits were down by more than a third as compared to before the pandemic. This reduction in patient visits was not unique for CSU. In fact, outpatient visits of psoriasis patients were reduced even more, by more than two thirds. Importantly, male CSU patient visit numbers were as affected as those in females, slightly less actually, suggesting that their CSU deterioration during the pandemic did not occur primarily because they did not visit their physicians.

That male but not female CSU patients experienced loss of disease control during the pandemic is likely linked to differences in their omalizumab treatment rates during as compared to before the pandemic and differences in omalizumab efficacy. As for omalizumab treatment rates, one of two male CSU patients used omalizumab during the pandemic, and this was the same as before the pandemic. Female patients more often used omalizumab during the pandemic than before the pandemic, and their levels of disease activity and rates of controlled disease remained stable during the pandemic. This suggests that the pandemic, overall, aggravates CSU and increases CSU disease activity. In female patients, increased use of omalizumab comes with unchanged rates of controlled disease. In male patients, unchanged use of omalizumab comes with reduced rates of controlled disease. Interestingly, female patients with psoriasis, like female patients with CSU, also used biologics more frequently during the pandemic as compared to before the pandemic, and male patients with psoriasis, like male patients with CSU, also did not increase their use. But in psoriasis, both male and female patients showed similar rates of decreased disease control during the pandemic. It is tempting to speculate that this points to a higher impact of the pandemic on psoriasis in female patients, who experience similar rates of worsening as male patients, despite using biologics markedly more often. In any case, the situation for male CSU patients during the pandemic appears to be worse not only compared to female CSU patients but also compared to male patients with psoriasis. In male patients with psoriasis, rates of controlled disease during the pandemic dropped by 15% as compared to more than 50% in male CSU patients, with unchanged rates of biological treatment in both. The first likely reason for the loss of disease control in male but not female patients with CSU is that the pandemic translates to higher skin mast cell activation, driving increased symptom occurrence and disease activity across all patients with CSU, male and female. This is mitigated, in the female CSU patient population, by the higher number of patients treated with omalizumab. In the male patient population, rates of omalizumab use are unchanged, and, consequently, loss of disease control is more frequent.

The second difference between male and female CSU patients linked to loss of disease control in the former but not the latter is the ability of omalizumab to control CSU. Only 50% of male omalizumab-treated patients reported controlled disease during the pandemic as compared to 92% before the pandemic, whereas two thirds of female omalizumab-treated patients experienced disease control during and before the pandemic. Why is the efficacy of omalizumab treatment, during the pandemic, reduced in male patients but not female patients with CSU? Our study does not and cannot answer this question. The least likely explanation is that the pharmacological effects of omalizumab, i.e. neutralization of IgE and downregulation of IgE receptors, are impaired by the pandemic, in male patients only. We cannot think of a mechanism that would explain this.

In addition, some of the effects of the pandemic that increase disease activity in CSU patients may be more prominent in males than in females. COVID-19 can exacerbate CSU, and it often does, in 3 of 10 affected patients and in 7 of 10 patients hospitalized because of COVID-19 (3). Did our male CSU patients have higher rates of COVID-19 than our female patients? Until August 2020, none of our CSU patients had been diagnosed with COVID-19 disease, however, we know that the incidence of COVID-19 at that time was 26.1 per 100,000 and the female:male ratio was 49:51^2^. This makes it unlikely that higher rates of CSU exacerbation in our male patients were due to higher rates of COVID-19. Pandemic-associated stress and anxiety, rather than COVID-19 itself, may have been more pronounced in males *vs* females. The pandemic comes with increased levels of stress in the general population, which is held to be linked to the fear of becoming infected, of financial hardship due to lockdowns or getting sick, of family members getting COVID-19, and of dying from COVID-19 (14). A recent study investigating the psychological burden of the pandemic on the CSU showed increased CSU activity during quarantine periods (15). On the date we base our study on (August 2020), our country had just come out of the first quarantine period. In our patient population, males are more often than females the breadwinners and providers of their families, and they also have higher numbers of social contacts and therefore risk of infection, so stress may have been more pronounced in males than females, and stress is an important driver of CSU disease activity. We acknowledge that this explanation is highly speculative and calls for further studies on pandemic-associated stress levels in males and females and their relevance for changes in CSU disease activity. Finally, male bias towards severe disease is a consistent feature of COVID-19 (16). It is, therefore, possible, that male COVID-19 patients with CSU experience disease exacerbation more often than female patients and that these exacerbations are more severe because of a more severe course of COVID-19. Interestingly, more severe COVID-19 in male patients as compared to female patients has been linked to differences in both the innate and adaptive immune system including T and B cell responses. Male patients produce less type 1 interferon (IFN), a potent anti-viral cytokine that is encoded, like many immune-related genes and regulatory elements involved in anti-viral immune responses, on the X chromosome (17, 18). Of note, type 1 IFN is known to suppress mast cell function, including histamine release, and poor IFN responses may, therefore, be linked to increased mast cell activation, which drives disease activity in patients with CSU (19).

Our study has several strengths and limitations. The strengths of this study include its sizeable patient numbers, the analysis of psoriasis as a control, and the fact that it was performed at an UCARE, the only in the region, making it the key referral center for patients with moderate or severe CSU. The limitations are that it was a single center and its retrospective design.

Taken together, our study demonstrates that the ongoing COVID-19 pandemic has a major impact on patients with CSU and psoriasis, and that this impact can differ across disease populations. Male but not female CSU patients, during the COVID-19 pandemic, show loss of disease control. The clinical learning from our findings is that the pandemic must be expected to increase the pathogenic drive and disease activity in CSU and other chronic inflammatory diseases. When this is met with increased use of effective treatment, rates of controlled disease are maintained. When the treatment remains unchanged, control is at risk of being lost.

## Data Availability Statement

The raw data supporting the conclusions of this article will be made available by the authors, without undue reservation.

## Author Contributions

RE and HK performed statistical analysis and drafted the manuscript. AA, HK, and MRA were involved in patient recruitment and proof-reading of the manuscript. MM, MA, and KÖ were involved in study planning and proof-reading of the manuscript. RE, HK, and MM planned the study, coordinated the study, collected patient data, was involved in statistical analysis and drafted the manuscript. All authors contributed to the article and approved the submitted version.

## Funding

This work was supported by intramural funding.

## Conflict of Interest

MM is or recently was a speaker and/or advisor for and/or has received research funding from Allakos, Amgen, Aralez, ArgenX, AstraZeneca, Celldex, Centogene, CSL Behring, FAES, Genentech, GIInnovation, Gilead, Innate Pharma, Kyowa Kirin, Leo Pharma, Lilly, Menarini, Moxie, Novartis, Roche, Sanofi/Regeneron, Third HarmonicBio, UCB, and Uriach. RE recently was a speaker for Novartis.

The remaining authors declare that the research was conducted in the absence of any commercial or financial relationships that could be construed as a potential conflict of interest.

The handling editor declared a past collaboration with one of the authors MM.

## Publisher’s Note

All claims expressed in this article are solely those of the authors and do not necessarily represent those of their affiliated organizations, or those of the publisher, the editors and the reviewers. Any product that may be evaluated in this article, or claim that may be made by its manufacturer, is not guaranteed or endorsed by the publisher.

## Footnotes

1 WHO Director-General’s opening remarks at the media briefing on COVID-19 - 5 March 2020 Available from: https://www.who.int/dg/speeches/detail/who-director-general-s-opening-remarks-at-the-media-briefing-on-covid-19---5-march-2020 [Accessed October 10, 2020].

2 Republic of Turkey Ministry of Health Weekly Case Numbers https://covid19.saglik.gov.tr/TR-68640/haftalik-rapor--weekly-report.html [Accessed April 10, 2021].

## Abbreviations

ACARE, Angioedema Centers of Reference and Excellence; CU, Chronic Urticaria; CSDU, Chronic Skin Diseases Unit; CSU, Chronic Spontaneous Urticaria; DLQI, Dermatology Life Quality Index; IQR, interquartile range; PASI, Psoriasis Area and Severity Index; UAS, Urticaria Activity Score; UCARE, Urticaria Center of Reference and Excellence; UCT, Urticaria Control Test.
